# Deubiquitinases at the interplay between hematopoietic stem cell aging and myelodysplastic transformation

**DOI:** 10.1002/1873-3468.14991

**Published:** 2024-08-06

**Authors:** Elisabetta Citterio, Antonella Ellena Ronchi

**Affiliations:** ^1^ Department of Biotechnology and Biosciences University of Milano‐Bicocca 20126 Milan Italy

**Keywords:** Acute Myeloid Leukemia (AML), Aging, Deubiquitinases, Deubiquitinating enzymes (DUBs), Hematopoietic Stem Cells (HSC), Myelodysplastic Syndromes (MDS), Ubiquitination

## Abstract

Hematopoietic stem cells (HSC) maintain blood production throughout life. Nevertheless, HSC functionality deteriorates upon physiological aging leading to the increased prevalence of haematological diseases and hematopoietic malignancies in the elderly. Deubiquitinating enzymes (DUBs) by reverting protein ubiquitination ensure proper proteostasis, a key process in HSC maintenance and fitness.

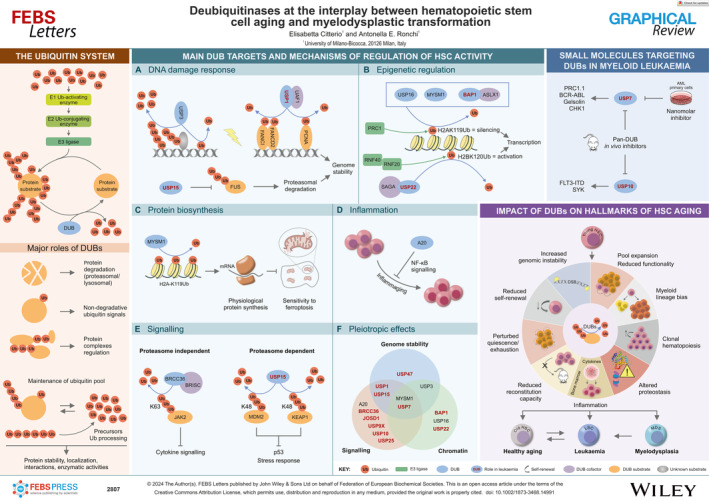

## Graphical abstract

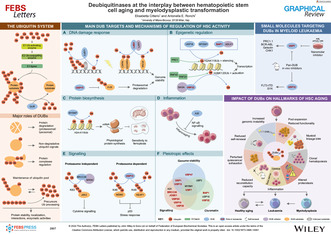

**Abbreviations**
AML: acute myeloid leukaemia
CML: chronic myelogenous leukaemia
DUB: deubiquitinating enzyme
H2AK119Ub: monoubiquitinated H2A
H2BK120Ub: monoubiquitinated H2B
HSC: hematopoietic stem cells
HSPC: hematopoietic stem and progenitor cells
KO: knockout
LSC: leukemic stem cells
MDS: myelodysplastic syndrome
MonoUb: monoubiquitinated
Ub: ubiquitin.

Hematopoietic stem cells (HSC) maintain blood production throughout life. Nevertheless, HSC functionality deteriorates upon physiological aging leading to the increased prevalence of haematological diseases and hematopoietic malignancies in the elderly. Deubiquitinating enzymes (DUBs) by reverting protein ubiquitination ensure proper proteostasis, a key process in HSC maintenance and fitness.

## The ubiquitin system

Ubiquitination is a reversible post‐translational modification involved in the regulation of most cellular processes. Ubiquitin (Ub), a 76 amino acid polypeptide, is primarily conjugated to lysine residues of substrate proteins through the sequential activity of E1‐activating, E2‐conjugating and E3 ligating (Ub‐ligase) enzymes, generating a complex code written on thousands of proteins [1].

## Major roles of deubiquitinating enzymes (DUBs)

DUBs catalyse the removal of ubiquitin from substrates, maintaining the balance between ubiquitination and deubiquitination [2]. The 100 human DUBs have been categorized into seven structurally related superfamilies: the cysteine proteases ubiquitin‐specific proteases (USP), ovarian tumor proteases (OTU), ubiquitin C‐terminal hydrolases (UCH), Josephin/MDJ, MINDY and ZUFSP families as well as the JAMM/MPN+ family of metalloproteases [2]. Besides rescuing proteins from degradation, DUBs regulate protein function by cleaving and editing non‐degradative ubiquitin signals. Through these mechanisms, DUBs control proteostasis and cellular signalling networks [2, 3].

## Main DUB targets and mechanisms of regulation of HSC activity

In physiological aging as in Myelodysplastic Syndrome (MDS) and Acute Myeloid Leukaemia (AML), DUBs, including USP7, USP15, and A20, exhibit either increased or decreased expression [4, 5, 6, 7]. Moreover, in leukaemia, USP10, USP9X, BRCC36, and BAP1 participate in chromosomal translocation or are inactivated by somatic mutations [8, 9] (Table 1). Substrate analysis, genetic deletion and/or mutation of DUBs in mouse models, and data from human samples confirm a wide role of DUBs in biological functions relevant to both normal and malignant HSC (leukemic stem cells, LSC) [8]. This is achieved through engagement in different molecular mechanisms: DNA damage response [5, 10, 11, 12, 13, 14, 15], epigenetic drift [9, 15, 16, 17, 18, 19], protein biosynthesis [20], inflammation [21], and proteasome‐independent or dependent signalling [6, 11, 22, 23, 24, 25, 26, 27, 28, 29] (panels A‐E and Table 1). These processes ultimately impact signal transduction, cell cycle and differentiation. Individual DUBs are often involved in multiple pathways such as maintenance of genome stability, chromatin modifications and intra/extracellular cytokine and stress signalling. Many DUBs act on the same mechanism with pleiotropic effect (F).

**Table 1:** Mammalian DUBs with consolidated roles in hematopoietic stem cells and their implications in aging and myelodysplastic transformation.

DUB	Main target(s) and impact on HSC aging hallmarks and myelodysplastic transformation. *Role in MDS/myeloid leukemia ^Differential expression in aging (https://agingsignature.webhosting.rug.nl/)	Pan‐DUB inhibitors tested in AML	
*Ubiquitin specific proteases (USP)*			
^USP1	USP1 regulates monoUb FANCD2 Fanconi Anemia protein, promoting resistance to DNA cross‐linking chemotherapeutic agents [10]. USP1 deubiquitinates monoUb PCNA [10]. *USP1 inhibition promotes the degradation of ID1, suppressing the growth of AML cells [11].	C527; SJB2‐043; pimozide [11]	
^USP3	*Usp3* KO mice display enhanced monoUb H2A and H2B, lymphopenia, reduced HSC compartment, and decline in HSC repopulation potential upon aging [12]. USP3 regulates Ub‐dependent DNA damage signalling to protect HSC from genotoxic stress [12].		
USP7	*USP7 inhibition: a) upregulates Gelsolin to induce differentiation of MDS cells [33]; b) reduces the viability of primary AML cells and tumour burden *in vivo* in PDX models [13, 16]; c) delays MLL‐AF9‐induced leukaemia *in vivo* in human leukaemia xenografts [16]. *USP7 deubiquitinates and stabilises CHK1 in AML cells [13]. *USP7 interacts with PRC1.1 complex and its catalytic activity is required for PRC1.1 stability [16]. *USP7 deubiquitinates and stabilises BCR‐ABL to promote the survival of CML cells [22]. *USP7 is differential expressed in MDS [4].	P22077 [13, 16, 33] P5091 [22, 33] FT671 (nanomolar USP7 inhibitor) [16]	
^USP9X	*USP9X binds to FLT3‐ITD and its downregulation cooperates with WP1130 DUB inhibitor to promote FLT3‐ITD degradation and apoptosis of AML cells [23]. *USP9X deubiquitinates and stabilises ALKBH5 and promotes cell survival in AML cellular and murine models [34].	WP1130; G9 [23, 34].	
^USP10	*USP10 stabilises oncogenic FLT3‐ITD in AML [24]. *USP10 deubiquitinates and stabilises SKP2 enhancing the growth of CML xenografts *in vivo* [25]. *USP10 deubiquitinates and stabilises the tyrosine kinase SYK promoting the proliferation of SYK‐driven AML cells [26].	HBX19818, P22077; [24, 26] Spautin [25]	
USP15	*Usp15* KO compromises mHSC maintenance and reconstitution potential *in vivo* [5]. *USP15 stabilises FUS and safeguards genome integrity in HSC and LSC [5]. *USP15 stabilises KEAP1 and MDM2 to modulate redox and p53 signalling in AML cell [6]. *USP15 is overexpressed in hAML [5, 6] and differentially expressed in MDS [4].	USP15‐Inh [6]	
USP16/ Ubp‐M	USP16 deubiquitinates H2AK119Ub and regulates transcriptional programs in HSPC [17, 19]. Its KO is embryonic lethal [35]. Conditional KO impairs HSC cell cycle and differentiation [17], whereas trisomy in a model for Down's syndrome associates with reduced HSC self‐renewal [35].		
USP22	USP22 deubiquitinates H2BK120Ub to regulate the expression of inflammatory and immune responsive genes. Its KO induces cell‐intrinsic emergency haematopoiesis with increased HSC proliferation and extramedullary haematopoiesis [18]. *USP22 deubiquitinates and stabilises PU.1 and its KO facilitates transformation in Ras‐driven AML [36]. *USP22 stabilises SIRT1 in FLTD‐ITD AML cells [32].		
USP25	*USP25 deubiquitinates and stabilises BCR‐ABL in Philadelphia+ CML [27].	AZ‐1; AZ‐2 [27]	
USP47	* USP47 deubiquitinates and stabilises YB‐1 to promote DNA repair in CML [14].	P22077 [14]	
*Ovarian tumour proteases (OTU)*			
^A20	A20 expression is reduced in hHSC upon aging [7]. A20 hematopoietic KO enhances NF‐κB activation and IFN‐γ signalling resulting in loss of HSC quiescence [21]. A20 heterozygous deletion results in HSC pool expansion, decreased regenerative potential and myeloid‐biased haematopoiesis in mice [7]. *A20 confers proliferative advantage to TLR‐TRAF6 primed MDS HSPC [8].		
*Ubiquitin C‐terminal hydrolases (UCH)*			
BAP1	*BAP1deubiquitinates the cell cycle regulator HCF‐1 and its binding partner OGT1 to suppress proliferation and cell cycle progression, limiting myeloid transformation [9]. *Mutant ASXL1‐MT/BAP1 complex promotes leukaemia transformation [8, 32].		
*Josephin/MDJ*		
JOSD1	*JOSD1 interacts with and stabilises JAK2‐V617F mutant to promote AML cells survival [29].	SB1‐F‐70; XL‐9872‐106C [29]
*JAMM/MPN+ metalloproteases*		
BRCC36 (BRCC3)	Deficiency of JAK2 K63‐polyUb deubiquitination by BRCC36, as part of the BRISC complex, stabilises JAK2 signalling, promoting HSC expansion [28]. *BRCC36 is mutated in MDS and AML [8].	
MYSM1	MYSM1 deubiquitinates H2AK119Ub, regulating transcriptional programs in HSPC [15, 19]. Inactivating *MYSM1* mutations cause an inherited bone marrow failure syndrome (IBMFS) [15]. MYSM1‐deficient HSC exhibit increased oxidative stress [15, 20], γH2AX DNA damage mark, ribosomal stress and p53 activation [15, 37]. By sustaining protein synthesis, MYSM1 protects hHSCs from ferroptosis [20]. Deficiency of MYSM1 activity causes loss of HSC quiescence and depletion of hematopoietic lineages [15, 38].	

## DUBs in hallmarks of HSC aging and myelodysplastic transformation

The same biological processes mentioned earlier contribute to key features observed in old HSC which are shared by LSC in age‐related pathologies such as MDS and AML [30, 31]. DUB knockout mice display features of premature aging HSC including perturbed quiescence (i.e. A20 [21]), reduced self‐renewal and reconstitution capacity (i.e. USP3 [12], USP15 [5], USP16 [17]), pool expansion and myeloid lineage bias (i.e. A20 [7], USP22 [18], BAP1 [9]), inflammation (A20 [21]), increased genomic instability (i.e. USP1 [10], USP3 [12], USP15 [5]), epigenetic changes (i.e. USP16 [17], USP22 [18], BAP1 [9], MYSM1 [15, 19]) and clonal hematopoiesis (BRCC36 [28]). Given their mechanistic pleiotropic effect, DUBs often impact more than one aspect and have partially overlapping roles in HSC fitness (Table 1).

## Small molecules targeting DUBs in myeloid leukaemia

Several DUB inhibitors small molecules have been developed and tested in *in vitro* and *in vivo* models of myeloid leukaemia, where their inhibition promotes proteasomal degradation of oncogenic proteins (such as FLT3‐ITD upon USP10 inhibition [24]) [22, 23, 26, 27, 29](Table 1). First‐generation compounds, such as P22077, have largely non‐specific pan‐DUB inhibitory activity [3]. The new class of small molecules, like the USP7 inhibitor FT671, shows high specificity with efficacy in primary AML cells at nanomolar concentration [16].

## Conclusion remarks and future perspective

DUBs dysfunction exacerbates HSC aging and predisposes them to leukaemia. As such, they are attractive therapeutic targets. New generation, structure‐based selective inhibitors are currently under evaluation in pre‐clinical models alone or in combinational therapies to overcome resistance and relapse [3, 32]. The translation of DUB inhibition into clinics will require meeting the parallel challenge of selecting key DUBs ‐ and their substrates ‐ that sustain aberrant cell proliferation in the specific leukaemia subtypes.

## References

[1] Dikic, I. & Schulman, B. A. (2023) An expanded lexicon for the ubiquitin code, Nat Rev Mol Cell Biol 24, 273–287. 36284179 10.1038/s41580-022-00543-1 PMC9595094

[2] Clague, M. J. , Urbe, S. & Komander, D. (2019) Breaking the chains: deubiquitylating enzyme specificity begets function, Nat Rev Mol Cell Biol 20, 338–352. 30733604 10.1038/s41580-019-0099-1

[3] Harrigan, J. A. , Jacq, X. , Martin, N. M. & Jackson, S. P. (2018) Deubiquitylating enzymes and drug discovery: emerging opportunities, Nat Rev Drug Discov 17, 57–78. 28959952 10.1038/nrd.2017.152 PMC7097658

[4] de Carvalho, L. G. A. , Komoto, T. T. , Moreno, D. A. , Goes, J. V. C. , de Oliveira, R. T. G. , de Lima Melo, M. M. , Roa, M. , Goncalves, P. G. , Montefusco‐Pereira, C. V. , Pinheiro, R. F. & Ribeiro Junior, H. L. (2023) USP15‐USP7 Axis and UBE2T Differential Expression May Predict Pathogenesis and Poor Prognosis in De Novo Myelodysplastic Neoplasm, Int J Mol Sci. 24. 10.3390/ijms241210058 PMC10298103 37373211

[5] van den Berk, P. , Lancini, C. , Company, C. , Serresi, M. , Sanchez‐Bailon, M. P. , Hulsman, D. , Pritchard, C. , Song, J. Y. , Schmitt, M. J. , Tanger, E. , Popp, O. , Mertins, P. , Huijbers, I. J. , Jacobs, H. , van Lohuizen, M. , Gargiulo, G. & Citterio, E. (2020) USP15 Deubiquitinase Safeguards Hematopoiesis and Genome Integrity in Hematopoietic Stem Cells and Leukemia Cells, Cell Rep 33, 108533. 33378683 10.1016/j.celrep.2020.108533 PMC7788286

[6] Niederkorn, M. , Ishikawa, C. , K, M. H. , Bartram, J. , Stepanchick, E., J, R. B., A, E. C.‐C. , Bolanos, L. C. , Uible, E. , Choi, K. , Wunderlich, M. , Perentesis, J. P. , T, M. C. , Filippi, M. D . & Starczynowski, D. T. (2022) The deubiquitinase USP15 modulates cellular redox and is a therapeutic target in acute myeloid leukemia, Leukemia 36, 438–451. 34465865 10.1038/s41375-021-01394-z PMC8807387

[7] Smith, M. A. , Culver‐Cochran, A. E. , Adelman, E. R. , Rhyasen, G. W. , Ma, A. , Figueroa, M. E. & Starczynowski, D. T. (2020) TNFAIP3 Plays a Role in Aging of the Hematopoietic System, Front Immunol 11, 536442. 33224133 10.3389/fimmu.2020.536442 PMC7670064

[8] Barabino, S. M. L. , Citterio, E. & Ronchi, A. E. (2021) Transcription Factors, R‐Loops and Deubiquitinating Enzymes: Emerging Targets in Myelodysplastic Syndromes and Acute Myeloid Leukemia, Cancers (Basel). 13, Transcription Factors, R‐Loops and Deubiquitinating Enzymes: Emerging Targets in Myelodysplastic Syndromes and Acute Myeloid Leukemia. 10.3390/cancers13153753 PMC8345071 34359655

[9] Dey, A. , Seshasayee, D. , Noubade, R. , French, D. M. , Liu, J. , Chaurushiya, M. S. , Kirkpatrick, D. S. , Pham, V. C. , Lill, J. R. , Bakalarski, C. E. , Wu, J. , Phu, L. , Katavolos, P. , LaFave, L. M. , Abdel‐Wahab, O. , Modrusan, Z. , Seshagiri, S. , Dong, K. , Lin, Z. , Balazs, M. , Suriben, R. , Newton, K. , Hymowitz, S. , Garcia‐Manero, G. , Martin, F. , Levine, R. L. & Dixit, V. M. (2012) Loss of the tumor suppressor BAP1 causes myeloid transformation, Science 337, 1541–6. 22878500 10.1126/science.1221711 PMC5201002

[10] Kim, J. M. , Parmar, K. , Huang, M. , Weinstock, D. M. , Ruit, C. A. , Kutok, J. L. & D'Andrea, A. D. (2009) Inactivation of murine Usp1 results in genomic instability and a Fanconi anemia phenotype, Dev Cell 16, 314–20. 19217432 10.1016/j.devcel.2009.01.001 PMC3134285

[11] Mistry, H. , Hsieh, G. , Buhrlage, S. J. , Huang, M. , Park, E. , Cuny, G. D. , Galinsky, I. , Stone, R. M. , Gray, N. S. , D'Andrea, A. D. & Parmar, K. (2013) Small‐molecule inhibitors of USP1 target ID1 degradation in leukemic cells, Mol Cancer Ther 12, 2651–62. 24130053 10.1158/1535-7163.MCT-13-0103-T PMC4089878

[12] Lancini, C. , van den Berk, P. C. , Vissers, J. H. , Gargiulo, G. , Song, J. Y. , Hulsman, D. , Serresi, M. , Tanger, E. , Blom, M. , Vens, C. , van Lohuizen, M. , Jacobs, H. & Citterio, E. (2014) Tight regulation of ubiquitin‐mediated DNA damage response by USP3 preserves the functional integrity of hematopoietic stem cells, J Exp Med 211, 1759–77. 25113974 10.1084/jem.20131436 PMC4144738

[13] Cartel, M. , Mouchel, P. L. , Gotanegre, M. , David, L. , Bertoli, S. , Mansat‐De Mas, V. , Besson, A. , Sarry, J. E. , Manenti, S. & Didier, C. (2021) Inhibition of ubiquitin‐specific protease 7 sensitizes acute myeloid leukemia to chemotherapy, Leukemia 35, 417–432. 32447346 10.1038/s41375-020-0878-x PMC7245510

[14] Lei, H. , Xu, H. Z. , Shan, H. Z. , Liu, M. , Lu, Y. , Fang, Z. X. , Jin, J. , Jing, B. , Xiao, X. H. , Gao, S. M. , Gao, F. H. , Xia, L. , Yang, L. , Liu, L. G. , Wang, W. W. , Liu, C. X. , Tong, Y. , Wu, Y. Z. , Zheng, J. K. , Chen, G. Q. , Zhou, L. & Wu, Y. L. (2021) Targeting USP47 overcomes tyrosine kinase inhibitor resistance and eradicates leukemia stem/progenitor cells in chronic myelogenous leukemia, Nat Commun 12, 51. 33397955 10.1038/s41467-020-20259-0 PMC7782553

[15] Fiore, A. , Liang, Y. , Lin, Y. H. , Tung, J. , Wang, H. , Langlais, D. & Nijnik, A. (2020) Deubiquitinase MYSM1 in the Hematopoietic System and beyond: A Current Review, Int J Mol Sci 21. 10.3390/ijms21083007 PMC7216186 32344625

[16] Maat, H. , Atsma, T. , Hogeling, S. , Rodríguez López, A. , Jaques, J. , Olthuis, M. , de Vries, M. , Gravesteijn, C. , Brouwers‐Vos, A. , van der Meer, N. , Datema, S. , Salzbrunn, J. , Huls, G. , Baas, R. , Martens, J. , van den Boom, V. & Schuringa, J. (2021) The USP7‐TRIM27 axis mediates non‐canonical PRC1.1 function and is a druggable target in leukemia, iScience. 24. 10.1016/j.isci.2021.102435 PMC8169803 34113809

[17] Gu, Y. , Jones, A. E. , Yang, W. , Liu, S. , Dai, Q. , Liu, Y. , Swindle, C. S. , Zhou, D. , Zhang, Z. , Ryan, T. M. , Townes, T. M. , Klug, C. A. , Chen, D. & Wang, H. (2016) The histone H2A deubiquitinase Usp16 regulates hematopoiesis and hematopoietic stem cell function, Proc Natl Acad Sci U S A 113, E51‐60. 26699484 10.1073/pnas.1517041113 PMC4711844

[18] Dietlein, N. , Wang, X. , Metz, J. , Disson, O. , Shang, F. , Beyersdorffer, C. , Rodriguez Correa, E. , Lipka, D. B. , Begus‐Nahrmann, Y. , Kosinsky, R. L. , Johnsen, S. A. , Lecuit, M. , Hofer, T. & Rodewald, H. R. (2022) Usp22 is an intracellular regulator of systemic emergency hematopoiesis, Sci Immunol 7, eabq2061. 36490327 10.1126/sciimmunol.abq2061

[19] Wang, H. , Langlais, D. & Nijnik, A. (2023) Histone H2A deubiquitinases in the transcriptional programs of development and hematopoiesis: a consolidated analysis, Int J Biochem Cell Biol 157, 106384. 36738766 10.1016/j.biocel.2023.106384

[20] Zhao, J. , Jia, Y. , Mahmut, D. , Deik, A. A. , Jeanfavre, S. , Clish, C. B. & Sankaran, V. G. (2023) Human hematopoietic stem cell vulnerability to ferroptosis, Cell 186, 732–747 e16, 732, 747.e16. 36803603 10.1016/j.cell.2023.01.020 PMC9978939

[21] Nakagawa, M. M. , Thummar, K. , Mandelbaum, J. , Pasqualucci, L. & Rathinam, C. V. (2015) Lack of the ubiquitin‐editing enzyme A20 results in loss of hematopoietic stem cell quiescence, J Exp Med 212, 203–16. 25624445 10.1084/jem.20132544 PMC4322050

[22] Jiang, S. , Wang, X. , He, Y. , Huang, H. , Cao, B. , Zhang, Z. , Liu, J. , Wang, Q. , Huang, Z. & Mao, X. (2021) Suppression of USP7 induces BCR‐ABL degradation and chronic myelogenous leukemia cell apoptosis, Cell Death Dis 12, 456. 33963175 10.1038/s41419-021-03732-6 PMC8105359

[23] Akiyama, H. , Umezawa, Y. , Ishida, S. , Okada, K. , Nogami, A. & Miura, O. (2019) Inhibition of USP9X induces apoptosis in FLT3‐ITD‐positive AML cells cooperatively by inhibiting the mutant kinase through aggresomal translocation and inducing oxidative stress, Cancer Lett 453, 84–94. 30946869 10.1016/j.canlet.2019.03.046

[24] Weisberg, E. L. , Schauer, N. J. , Yang, J. , Lamberto, I. , Doherty, L. , Bhatt, S. , Nonami, A. , Meng, C. , Letai, A. , Wright, R. , Tiv, H. , Gokhale, P. C. , Ritorto, M. S. , De Cesare, V. , Trost, M. , Christodoulou, A. , Christie, A. , Weinstock, D. M. , Adamia, S. , Stone, R. , Chauhan, D. , Anderson, K. C. , Seo, H. S. , Dhe‐Paganon, S. , Sattler, M. , Gray, N. S. , Griffin, J. D. & Buhrlage, S. J. (2017) Inhibition of USP10 induces degradation of oncogenic FLT3, Nat Chem Biol 13, 1207–1215. 28967922 10.1038/nchembio.2486 PMC6314479

[25] Liao, Y. , Liu, N. , Xia, X. , Guo, Z. , Li, Y. , Jiang, L. , Zhou, R. , Tang, D. , Huang, H. & Liu, J. (2019) USP10 modulates the SKP2/Bcr‐Abl axis via stabilizing SKP2 in chronic myeloid leukemia, Cell Discov 5, 24. 31044085 10.1038/s41421-019-0092-z PMC6488640

[26] Yang, J. , Meng, C. , Weisberg, E. , Case, A. , Lamberto, I. , Magin, R. S. , Adamia, S. , Wang, J. , Gray, N. , Liu, S. , Stone, R. , Sattler, M. , Buhrlage, S. & Griffin, J. D. (2020) Inhibition of the deubiquitinase USP10 induces degradation of SYK, Br J Cancer 122, 1175–1184. 32015510 10.1038/s41416-020-0731-z PMC7156412

[27] Shibata, N. , Ohoka, N. , Tsuji, G. , Demizu, Y. , Miyawaza, K. , Ui‐Tei, K. , Akiyama, T. & Naito, M. (2020) Deubiquitylase USP25 prevents degradation of BCR‐ABL protein and ensures proliferation of Ph‐positive leukemia cells, Oncogene 39, 3867–3878. 32203161 10.1038/s41388-020-1253-0

[28] Donaghy, R. , Han, X. , Rozenova, K. , Lv, K. , Jiang, Q. , Doepner, M. , Greenberg, R. A. & Tong, W. (2019) The BRISC deubiquitinating enzyme complex limits hematopoietic stem cell expansion by regulating JAK2 K63‐ubiquitination, Blood 133, 1560–1571. 30755420 10.1182/blood-2018-10-877563 PMC6450430

[29] Yang, J. , Weisberg, E. L. , Liu, X. , Magin, R. S. , Chan, W. C. , Hu, B. , Schauer, N. J. , Zhang, S. , Lamberto, I. , Doherty, L. , Meng, C. , Sattler, M. , Cabal‐Hierro, L. , Winer, E. , Stone, R. , Marto, J. A. , Griffin, J. D. & Buhrlage, S. J. (2022) Small molecule inhibition of deubiquitinating enzyme JOSD1 as a novel targeted therapy for leukemias with mutant JAK2, Leukemia 36, 210–220. 34326465 10.1038/s41375-021-01336-9

[30] Yamashita, M. , Dellorusso, P. V. , Olson, O. C. & Passegue, E. (2020) Dysregulated haematopoietic stem cell behaviour in myeloid leukaemogenesis, Nat Rev Cancer 20, 365–382. 32415283 10.1038/s41568-020-0260-3 PMC7658795

[31] Mejia‐Ramirez, E. , Geiger, H. & Florian, M. C. (2020) Loss of epigenetic polarity is a hallmark of hematopoietic stem cell aging, Hum Mol Genet 29, R248‐R254. 32821941 10.1093/hmg/ddaa189

[32] Dewson, G. , Eichhorn, P. J. A. & Komander, D. (2023) Deubiquitinases in cancer, Nat Rev Cancer, 23, 842, 862 37935888 10.1038/s41568-023-00633-y

[33] Deng, J. , Liang, L. , Yi, H. , Su, T. , Yang, Z. , Nie, L. & Liu, J. (2020) USP7 inhibition inhibits proliferation and induces megakaryocytic differentiation in MDS cells by upregulating gelsolin, Br J Haematol 190, 418–429. 32130729 10.1111/bjh.16549

[34] Wang, P. , Wang, J. , Yao, S. , Cui, M. , Cheng, Y. , Liu, W. , Gao, Z. , Hu, J. , Zhang, J. & Zhang, H. (2023) Deubiquitinase USP9X stabilizes RNA m(6)A demethylase ALKBH5 and promotes acute myeloid leukemia cell survival, J Biol Chem 299, 105055. 37454738 10.1016/j.jbc.2023.105055 PMC10424212

[35] Adorno, M. , Sikandar, S. , Mitra, S. S. , Kuo, A. , Nicolis Di Robilant, B. , Haro‐Acosta, V. , Ouadah, Y. , Quarta, M. , Rodriguez, J. , Qian, D. , Reddy, V. M. , Cheshier, S. , Garner, C. C. & Clarke, M. F. (2013) Usp16 contributes to somatic stem‐cell defects in Down's syndrome, Nature 501, 380–4. 24025767 10.1038/nature12530 PMC3816928

[36] Melo‐Cardenas, J. , Xu, Y. , Wei, J. , Tan, C. , Kong, S. , Gao, B. , Montauti, E. , Kirsammer, G. , Licht, J. D. , Yu, J. , Ji, P. , Crispino, J. D. & Fang, D. (2018) USP22 deficiency leads to myeloid leukemia upon oncogenic Kras activation through a PU.1‐dependent mechanism, Blood 132, 423–434. 29844011 10.1182/blood-2017-10-811760 PMC6071563

[37] Belle, J. I. , Wang, H. , Fiore, A. , Petrov, J. C. , Lin, Y. H. , Feng, C. H. , Nguyen, T. T. M. , Tung, J. , Campeau, P. M. , Behrends, U. , Brunet, T. , Leszinski, G. S. , Gros, P. , Langlais, D. & Nijnik, A. (2020) MYSM1 maintains ribosomal protein gene expression in hematopoietic stem cells to prevent hematopoietic dysfunction, JCI Insight 5. 10.1172/jci.insight.125690 PMC7406308 32641579

[38] Liang, Y. , Bhatt, G. , Tung, L. T. , Wang, H. , Kim, J. E. , Mousa, M. , Plackoska, V. , Illes, K. , Georges, A. A. , Gros, P. , Henneman, L. , Huijbers, I. J. , Nagar, B. & Nijnik, A. (2023) Deubiquitinase catalytic activity of MYSM1 is essential in vivo for hematopoiesis and immune cell development, Sci Rep 13, 338. 36611064 10.1038/s41598-023-27486-7 PMC9825392

